# The Effect of Religious Coping Levels on Asthma Control in Asthmatic Adults in Türkiye

**DOI:** 10.1007/s10943-025-02360-0

**Published:** 2025-06-16

**Authors:** Canan Bozkurt, Hülya Bulut

**Affiliations:** 1 Department of Internal Medicine Nursing, Nursing Department, Faculty of Health Sciences, Bandirma Onyedi Eylul University, Yeni District, Şehit Astsubay Mustafa Soner Varlık Street, No: 77, 10250 Bandirma, Balıkesir Turkey; 2 University of Health Sciences Dr. Suat Seren Chest Diseases and Thoracic Surgery Training and Research Hospital, Izmir, Turkey

**Keywords:** Asthma control, Asthmatic adults, Religious coping, Positive religious coping, Negative religious coping

## Abstract

This study examines the effect of religious coping on asthma control in asthmatic adults. The sample included 500 adults who visited outpatient clinics in western Türkiye between April 24 and October 30, 2024. The Patient Identification Form, Religious Coping Scale (RCS), and Asthma Control Test (ACT) were used. The mean ACT score was 14.27 ± 3.81, with 81.6% of participants having uncontrolled asthma (ACT score ≤ 19). Multiple regression analysis revealed that positive religious coping was positively associated with better asthma control (*B* = 0.287, *p* < 0.001), while negative religious coping was negatively associated with asthma control (*B* =  − 0.378, *p* < 0.001). The explanatory power of religious coping on asthma control was 15.4% (Adjusted *R*^2^ = 0.154, *p* < 0.001). The indings highlight religious coping as a significant psychosocial support mechanism in asthma management, suggesting its potential to improve patient quality of life. Healthcare providers, especially nurses, should integrate coping support in asthma education.

## Introduction

Asthma, which affects 339 million people worldwide and is estimated to cause 455,000 deaths, is the most common chronic inflammatory airway disease (Vos et al., 2020). Asthma can affect people of all ages, usually beginning in childhood, with prevalence increasing with age (World Health Organization [WHO], 2024). While asthma prevalence ranges between 5 and 10%, the European Respiratory Society highlights the difficulty of diagnosing asthma in adults due to the use of inadequate or excessive diagnostic methods (Global Initiative for Asthma [GINA], 2024; Louis et al., 2022). Furthermore, more than 80% of asthma-related deaths occur in low- and middle-income countries (GINA, 2024)

Asthma is marked by key symptoms, including shortness of breath, wheezing, chest tightness, and coughing, along with fluctuating expiratory airflow (GINA, 2024; Bloom et al., 2019). These symptoms can disrupt daily activities and negatively affect quality of life. Worsening asthma symptoms can cause sleep disturbances, daytime fatigue, and difficulties in school or work attendance. Effective management requires consistent medication use, avoidance of triggers, and lifestyle adjustments (WHO, 2024; Beasley et al., 2020). Proper asthma control is crucial to prevent permanent airway damage, enhance quality of life, and reduce healthcare burdens (Ban et al., 2018).

Asthmatic adults must develop knowledge, confidence, and self-management skills to handle their condition (Bektaş et al., 2013; Wilson et al., 2010). Asthma management not only involves physiological care but also considers psychological and social factors (Adams et al., 2004; Sagmen et al., 2020). In particular, religious coping can serve as a critical psychosocial resource during asthma management, offering mental resilience and a framework for managing symptoms effectively.

Religious coping refers to seeking spiritual support through religious beliefs and practices during difficult life events. It can positively impact both physical and mental health, especially in long-term stress situations like chronic illnesses. These strategies enhance quality of life, reduce stress, and improve the mental well-being of patients and caregivers (Sarwoko et al., 2022). Religious coping also promotes hope and encourages a more positive outlook when managing illness (Krause & Ironson, 2024; Taskin Yilmaz et al., 2022). This approach is particularly significant in societies where religion plays an integral role in daily life and cultural identity, such as Türkiye. However, the way religious coping manifests can vary significantly across different cultural and religious contexts, which warrants further exploration to understand its nuances and broader implications.

In chronic lung diseases, spiritual support has been found to improve disease management, mental health, and quality of life (Duarte et al., 2020; Mesquita et al., 2022; Taskin Yilmaz et al., 2022). Positive religious coping strategies, such as praying, trusting in God, and adopting a positive perspective, provide psychological relief (Celik et al., 2022; Karatana & Yıldız, 2024). Conversely, negative religious coping, such as perceiving illness as divine punishment, can harm mental health (Sabanciogullari & Taskin Yilmaz, 2021).

Studies indicate that religious coping serves as a significant psychosocial support mechanism for individuals managing chronic diseases. Sagmen et al. (2020) found that “Turning to Religion and Planning” was a leading coping strategy among asthmatic adults. Similarly, Ruppe et al. (2024) reported that religiosity during young adulthood was linked to reduced depressive symptoms later in life among adolescents with asthma. Consistently, Adams et al. (2011) emphasized the importance of religious problem-solving in pediatric asthma management, demonstrating that God-involved and self-directed problem-solving strategies significantly influenced treatment adherence and parental coping among urban families. On the other hand, Ahmedani et al. (2013) found that belief in God’s control over health was associated with lower adherence to asthma controller medications in adult patients, highlighting the dual role of religiosity as both a supportive and challenging factor in chronic disease management.

Although limited research has explored the relationship between religion and asthma control (Adams et al., 2011; Ahmedani et al., 2013; Ruppe et al., 2024; Sagmen et al., 2020), no studies have specifically examined the role of religious coping in managing asthma. Understanding the effect of religious coping on asthma control may inform new strategies to enhance the quality of life for asthmatic adults. In Türkiye, where religious beliefs and practices hold cultural significance, further research into the role of spiritual support could provide valuable insights.

## Methods

### Aim

The aim of this study is to examine the relationship between positive and negative religious coping scores and asthma control in asthmatic adults and to determine the explanatory power of these coping strategies in asthma control.

### Research Questions

- How do positive and negative religious coping scores affect asthma control in asthmatic adults?
- What other factors influence asthma control in asthmatic adults?

### Type of the Study

This descriptive and cross sectional study was prepared in accordance with the STrengthening the Reporting of OBservational studies in Epidemiology (STROBE) guidelines.

### Population and Sample

The population of the study consisted of adults aged 18 and older who had been diagnosed with asthma and had applied to the outpatient clinics of a chest diseases and surgery training and research hospital in western Türkiye. In 2023, the number of patients who were diagnosed with asthma and applied to the outpatient clinics of the hospital was 3,200. The sample size calculation for the known population was performed using www.openepi.com, and with a 95% confidence interval and a ± 5% margin of error, the minimum suitable sample size was determined to be 344. However, to increase generalizability, the study was completed with 500 patients. A total of 570 patients were approached, and 500 agreed to participate, resulting in a response rate of 87.7%. A power analysis conducted using the G*Power 3.1.9.2 program after the study found the effect size (*ρ*) to be 0.351, the standard error (*α*) to be 0.05, and the power of the study (1 − *β*) to be 100%.

The study included patients aged 18 and older who were diagnosed with asthma by a physician and were able to communicate in Turkish. Patients with a psychiatric diagnosis by a physician, those who refused to participate, or those who provided incomplete responses were excluded from the study.

### Data Collection Tools

#### Individual Information Form

The form was created by the researchers and contains a total of 13 questions. Six questions pertain to sociodemographic information (age, gender, marital status, education level, employment status, and place of residence), and seven questions relate to health and disease (smoking habits, years with asthma, number of hospital visits due to asthma attacks, history of hospitalization due to asthma, etc.).

#### Religious Coping Scale (RCS)

The scale developed by Abu Raiya et al. (2008), consists of 10 items and two subscales (positive and negative). Seven items measure positive religious coping, while three items measure negative religious coping. The scale uses a four-point Likert measurement, ranging from “1” (I almost never do this) to “4” (I do this often). The scale does not provide a total religious coping score; instead, the scores for positive and negative religious coping are calculated separately. Scores on the positive religious coping scale range from 7 to 28, while scores on the negative religious coping scale range from 3 to 12. High positive coping scores indicate constructive religious coping, while high negative coping scores indicate maladaptive religious coping. In the Turkish validity and reliability study conducted by Ekşi and Sayın (2016), the 10-item, 2-factor structure was deemed acceptable. The Cronbach’s alpha internal consistency coefficient was 0.90 for the entire scale, 0.86 for the negative subscale, and 0.91 for the positive subscale. In our study, the Cronbach’s alpha was 0.88 for the entire scale, 0.89 for the positive subscale, and 0.81 for the negative subscale.

To provide a clearer understanding for readers unfamiliar with the scale, examples of items include the following: from the positive religious coping subscale—“When I face a problem in life, I try to get closer to God”, “I consider it a test from God to deepen my faith”, and “I seek God’s love and protection”; and from the negative religious coping subscale—“When I face a problem in life, I believe I am being punished by God for my sins”, “I wonder what I have done to deserve God’s punishment”, and “I think I am being punished by God because I am not a faithful servant”.

#### Asthma Control Test (ACT)

The test developed by Nathan et al. (2004), is designed as a simple tool to identify uncontrolled asthma that can be easily administered by both patients and healthcare professionals. It is a five-question survey that evaluates asthma control based on criteria outlined in asthma guidelines, such as asthma symptoms, the need for rescue medication, and the impact of asthma on daily life. Each question is scored on a scale from zero to five. The highest possible score is 25, and the lowest is 5. An ACT score of 25 indicates full control, scores between 24 and 20 indicate partial control, and scores of ≤ 19 indicate poor control. The Turkish validity and reliability of the ACT were confirmed by Uysal et al. (2013). In the Turkish validity and reliability study, the Cronbach’s alpha value was found to be 0.84 (Uysal et al., 2013). In this study, however, the Cronbach’s alpha value for the ACT was determined to be 0.79.

#### Data Collection

After obtaining institutional and ethics committee approval, adults with asthma who visited the hospital’s outpatient clinics between April 24 and October 30, 2024, were informed about the study’s purpose and content. Participants who agreed to participate and met the inclusion criteria were asked to sign an informed consent form by a specialized nurse assistant researcher, and the questionnaires were distributed to them for self-completion. For individuals who were unable to fill out the questionnaires themselves (due to age, vision impairment, unwillingness, etc.), the researcher administered the questions, and each questionnaire took approximately 10–15 min to complete.

#### Ethical Principles of the Study

For the conduct of the study, preliminary institutional approval was obtained from the XXX Chest Diseases and Surgery Training and Research Hospital during a meeting on XX.XX.2024. Following the preliminary institutional approval, ethics committee approval was obtained from the XXX Non-Interventional Research Ethics Committee with the decision number XXX, dated XX.XX.2024. Informed consent forms were signed by the participants, and the study was conducted in accordance with the Declaration of Helsinki.

#### Data Analysis

The data obtained from the study were analyzed using the Statistical Package for Social Science (SPSS) version 23 on a dedicated computer. Means and standard deviations were calculated for the variables. Multiple linear regression (enter method) was used, and Asthma Control Test scores were considered dependent continuous variables. All variables were included as predictors in the multiple linear regression analysis. Categorical independent variables were converted into dummy variables. The Durbin–Watson test was used to assess whether the residuals in the linear regression models were independent. Model fit was evaluated using the coefficient of determination (R-squared). The significance level was set at *p* < 0.05.

## Results

The participants’ mean age was 65.60 ± 11.82, and 53.2% were aged 65 or older. Of the asthmatic adults, 63.8% were female, 80.2% were primary school graduates, 82% were married, and 59% were retired. A total of 78.7% of the participants perceived their income level as “moderate”, and 89% lived in a district or city center (Table 1).

**Table 1:** Descriptive characteristics of the participants

Variables	Total: 500 (100.0)
Age (years), *M* ± SD (min–max)	65.60 ± 11.82 (24–93)
Age groups, *n* (%)	
< 65 years	234 (46.8)
≥ 65 years	266 (53.2)
Gender, *n* (%)	
Female	319 (63.8)
Male	181 (36.2)
Education level, *n* (%)	
Primary school	401 (80.2)
Secondary school	55 (11.0)
High school and above	44 (8.8)
Marital status, *n* (%)	
Single	72 (18.0)
Married	428 (82.0)
Employment status, *n* (%)	
Employed	95 (19.0)
Unemployed	110 (22.0)
Retired	295 (59.0)
Perceived income status, *n* (%)	
Low	131 (26.2)
Middle	315 (63.0)
High	54 (10.8)
Place of residence,* n* (%)	
Rural	55 (11.0)
District/city center	445 (89.0)

*M* Mean; *SD* Standard deviation

Of the adults, 56.2% had quit smoking, while 15.8% continued to smoke. The average duration of asthma was 10.17 ± 5.94 years, and 51.2% had been diagnosed with asthma for more than 10 years. Among the participants, 86% reported using their medication regularly, 50.8% had received medication education, 55.6% had visited the hospital 1–2 times in the past year due to an asthma attack, and 56.4% stated that no one else in their family had been diagnosed with asthma (Table 2).

**Table 2:** Distribution of participants’ characteristics related to health and illness

Variables	Total: 500 (100.0)
Smoking habit, *n* (%)	
Never smokes	140 (28.0)
Smokes	79 (15.8)
Quit	281 (56.2)
Year of asthma, *M* ± SD (min–max)	10.17 ± 5.94 (1–22)
Asthma years groups, *n* (%)	
≤ 2 years	54 (10.8)
3–5 years	84 (16.8)
6–9 years	106 (21.2)
10–14 years	130 (26.0)
≥ 15 years	126 (25.2)
Regular use of medicines, *n* (%)	
Yes	430 (86.0)
No	70 (14.0)
Receiving medication training, *n* (%)	
Yes	254 (50.8)
No	246 (49.2)
Number of hospital admissions due to asthma attack, *n* (%)	
1–2 times	278 (55.6)
3–4 times	156 (31.2)
5 times ≤	66 (13.2)
Hospitalization for asthma attack, *n* (%)	
Yes	344 (68.8)
No	156 (31.2)
Family members diagnosed with asthma, *n* (%)	
Yes	218 (43.6)
No	282 (56.4)

The mean score for the Positive Religious Coping Scale was 18.47 ± 4.09, and for the Negative Religious Coping Scale, it was 4.95 ± 1.90. The average score for the Asthma Control Test (ACT) among asthmatic adults was 14.27 ± 3.81, with no participants achieving full control, and 81.6% of participants were found to have uncontrolled asthma (Table 3).

**Table 3:** Mean and distribution of religious coping scale and asthma control test scores

Variables	Mean ± SD (min–max)
Religious coping scale	
Positive religious coping	18.47 ± 4.09 (10–24)
Negative religious coping	4.95 ± 1.90 (3–12)
Asthma control test	14.27 ± 3.81 (6–23)
Asthma control, *n* (%)	500 (100.0)
Full control (25p)	0 (0.0)
Partial control (20p–24p)	92 (18.4)
Uncontrolled (≤ 19p)	408 (81.6)

*SD* Standard deviation

In the multiple regression analysis, according to Model 1, positive religious coping was a positive predictor of the Asthma Control Test (ACT) score, while negative religious coping was a negative predictor. The explanatory power of religious coping on the ACT was 15.4% (Adjusted *R*^2^: 0.154, *p* < 0.001). A one-unit increase in positive religious coping resulted in a 0.28-unit increase in ACT score (*B* = 0.287), while a one-unit increase in negative religious coping led to a 0.37-unit decrease in ACT score (*B* =  − 0.378) (*p* < 0.001). According to Model 2, being employed (*B* = 1.118), perceiving income as moderate (*B* = 3.059) or high (*B* = 7.586), receiving medication education (*B* = 0.679), and the level of positive religious coping (*B* = 0.191) were positive predictors of asthma control (*p* < 0.05). Male gender (*B* =  − 0.907), living in a district or city center (*B* =  − 1.686), smoking (*B* =  − 0.682), the number of hospital visits due to asthma attacks (*B* =  − 0.502), and negative religious coping (*B* =  − 0.272) were negative predictors of asthma control (*p* < 0.05). Age, education level, marital status, being retired, having quit smoking, duration of asthma, regular medication use, hospitalization due to an attack, and having a family member diagnosed with asthma did not affect asthma control (*p* > 0.05). The explanatory power of these independent variables on asthma control was found to be 56.9% (Adjusted *R*^2^: 0.569, *p* < 0.001) (Table 4).

**Table 4:** Multiple regression analysis of the relationship between participants’ descriptive characteristics and religious coping levels and asthma control test

Models. ACT	*B*	95.0% CI	*β*	*t*	*p* value
Model 1. ACT					
Positive religious coping scale	0.287	0.210, 0.364	0.308	7.297	< 0.001^*^
Negative religious coping scale	− 0.378	− 0.545, − 0.212	− 0.189	− 4.476	< 0.001^*^
	Adjust *R*^2^: 0.154, *F*: 46.261, *p* < 0.001^*^				
Model 2. ACT					
Positive religious coping scale	0.191	0.128, 0.254	0.118	2.817	< 0.001^*^
Negative religious coping scale	− 0.272	− 0.393, − 0.150	− 0.136	− 4.388	< 0.001^*^
Age	− 0.02	− 0.069, 0.028	− 0.063	− 0.819	0.413
Gender (ref. female)					
Male	− 0.907	− 1.443, − 0.372	− 0.115	− 3.329	0.001^**^
Education level (ref. Primary school)					
Secondary school	− 0.67	− 1.493, 0.153	− 0.05	− 1.601	0.4
High school and above	0.31	− 0.413, 1.033	0.026	0.843	0.11
Marital status (ref. Single)					
Married	0.09	− 0.598, 0.777	0.008	0.256	0.798
Employment status (ref. Retired)					
Employed	1.118	0.401, 1.836	0.115	3.064	0.002^**^
Unemployed	0.502	0.107, 1.111	0.055	1.62	0.106
Perceived income status (ref. Low)					
Middle	3.059	2.521, 3.597	0.389	11.178	< 0.001^*^
High	7.586	6.716, 8.455	0.621	17.147	< 0.001^*^
Place of recidence (ref. Rural)					
District/city center	− 1.686	− 2.460, − 0.912	− 0.139	− 4.281	< 0.001^* ^
Smoking habit (ref. Never smokes)					
Smokes	− 0.682	− 0.017, − 1.381	0.065	1.918	0.046^** ^
Quit	0.286	− 0.302, 0.873	0.034	0.956	0.34
Year of asthma	0.005	− 0.089, 0.100	0.008	0.114	0.91
Regular use of medicines (ref. No)					
Yes	0.326	− 0.347, 0.999	0.099	0.952	0.341
Receiving medication training (ref. No)					
Yes	0.679	0.222, 1.136	0.088	2.917	0.004^**^
Number of hospital admissions due to asthma attack	− 0.502	− 0.654, − 0.350	− 0.2	− 6.493	< 0.001^*^
Hospitalization for asthma attack (ref. No)					
Yes	0.083	− 0.371, 0.537	0.011	0.345	0.73
Family members diagnosed with asthma (ref. No)					
Yes	0.07	− 0.384, 0.524	0.009	0.296	0.767
	Adjust *R*^2^: 0.569, *F*: 33.756, *p* < 0.001^*^				

Bold values indicate statistically significant results ^*^p < 0.001, ^**^p < 0.05

*ACT* Asthma control test, *B* Unstandardized coefficients, *CI* Confidence interval, *β* Standardized regression coefficient, *R*^2^ Coefficient of determination

## Discussion

This study was conducted to examine the effect of religious coping levels on asthma control in asthmatic adults, with a total of 500 participants evaluated. The majority of participants were women, aged 65 and older, with low education levels, married, retired, perceiving their income as moderate, and living in a district or city center. These sociodemographic characteristics are consistent with findings from other studies conducted on asthmatic adults (Ndarukwa et al., 2020; Bozbaş et al., 2011).

The mean ACT score of the participants in the study was 14.27, and none achieved full asthma control. Approximately four-fifths of the participants had uncontrolled asthma, aligning with previous studies that highlight the global prevalence of uncontrolled asthma despite advances in pharmacological management and improved healthcare access (GINA, 2024; Ndarukwa et al., 2020; Bozbaş et al., 2011). This finding is particularly significant and can be linked to broader challenges in managing chronic diseases. Factors such as adherence to treatment, lifestyle modifications, and the need for consistent psychosocial support are critical barriers to achieving full asthma control. Şanlıtürk (2022) reported a higher rate of fully controlled asthma in a smaller study with 200 patients, suggesting that sample size and demographic variations may influence outcomes. In this context, larger-scale studies are needed to further investigate the complex dynamics affecting chronic disease management and the achievement of full control.

Participants had an average positive religious coping score of 18.47, indicating a moderate level, while their negative coping score averaged 4.95, reflecting a low level. Other studies on Turkish patients with COPD, fibromyalgia, and infertility reported higher positive religious coping scores and similar negative coping scores (Demirel et al., 2021; Taskin Yilmaz et al., 2022; Yüksel et al., 2022). This difference may be attributed to variations in disease characteristics, the perceived severity of the illness, or differences in cultural and personal attitudes toward religious coping. Patients with Parkinson’s disease, heart failure, diabetes, and cancer undergoing chemotherapy showed higher scores for both positive and negative religious coping (Çamcı et al., 2024; Celik et al., 2022; Karatana & Yıldız, 2024; Sabanciogullari & Taskin Yilmaz, 2021). This could be explained by the more severe or life-threatening nature of these conditions, which may intensify both positive reliance on spirituality and negative emotions such as guilt or questioning faith. In contrast, asthma, while chronic, might not invoke the same level of existential reflection, resulting in relatively lower coping scores. These comparisons underline the importance of context when interpreting religious coping levels and highlight the need for tailored psychosocial interventions based on the unique experiences and challenges of each patient group.

Our analysis found a positive but weak correlation between positive religious coping and asthma control, while negative religious coping showed a weak negative correlation. Additionally, in our study, a one-unit increase in positive coping improved asthma control by 0.28 units, whereas a one-unit increase in negative coping reduced asthma control by 0.37 units. Regarding asthma, Adams et al. (2011) emphasized the role of religious problem-solving in chronic disease management, particularly in urban families dealing with pediatric asthma, where coping strategies influenced both adherence and outcomes. Similarly, Ahmedani et al. (2013) highlighted the dual impact of religiosity, reporting that belief in God’s control could either support or hinder disease management depending on the context. Outside of asthma, Taskin Yilmaz et al. (2022) reported similar findings in patients with COPD, where positive religious coping was associated with increased hope, while negative coping was linked to reduced hope. These findings underscore the nuanced role of religious coping in chronic disease management, highlighting its potential to influence both positive and negative health outcomes. The observed parallels between asthma and COPD suggest that promoting positive religious coping strategies while addressing the detrimental effects of negative coping may improve overall disease management and patient well-being.

Sociodemographic factors also influenced asthma control. Being male was associated with a 0.90-unit decrease in asthma control, likely due to behavioral patterns such as lower healthcare-seeking behavior (Sagmen et al., 2020). Employment increased asthma control by 1.11 units, potentially due to higher physical activity and social engagement. Similarly, individuals perceiving their income as moderate or high had better control, with high-income participants experiencing a significant 7.58-unit increase in asthma control. This aligns with findings indicating that lower socioeconomic status correlates with higher anxiety, depression, and poorer asthma control (Sagmen et al., 2020). Ahmedani et al. (2013) also noted that financial constraints and low income could hinder effective asthma management due to reduced access to care.

Living in urban areas was identified as a negative predictor of asthma control, resulting in a 1.68-unit decrease. This finding aligns with existing evidence suggesting that urban environments pose unique challenges to effective asthma management. Factors such as air pollution, traffic emissions, and industrial waste are prevalent in cities and are known to exacerbate asthma symptoms by increasing airway inflammation and sensitivity (GINA, 2024). Furthermore, urban lifestyles often involve high levels of stress, crowded living conditions, and limited green spaces, all of which can negatively impact respiratory health and overall well-being. Access to healthcare in urban settings, while generally more abundant, may also be hindered by factors such as long wait times, high patient-to-provider ratios, and financial barriers (Sagmen et al., 2020). On the other hand, rural areas often offer fewer environmental triggers, such as lower levels of air pollution and reduced exposure to traffic-related emissions, potentially contributing to better asthma control (Louis et al., 2022). To understand the mechanisms underlying the urban–rural disparity in asthma control, future research could explore specific urban exposures, such as particulate matter (PM2.5 and PM10) and nitrogen dioxide (NO_2_), and their direct effects on lung function (Meo et al., 2024). Additionally, investigating the role of socioeconomic factors, healthcare accessibility, and urban stressors could provide further insights. The impact of lifestyle differences, including dietary habits, physical activity levels, and access to green spaces, may also warrant examination. Such research would contribute to a more comprehensive understanding of how urban living affects asthma outcomes and inform targeted interventions to improve asthma management in urban settings. Similarly, participants who continued smoking had a 0.68-unit decrease in ACT scores, further demonstrating the detrimental effects of modifiable environmental and behavioral factors, such as urban pollution and smoking, on asthma management (GINA, 2024; Beasley et al., 2020).

Education on medication use emerged as a positive predictor, improving asthma control by 0.67 units. Patient education through guided management plans and follow-ups has been shown to enhance asthma outcomes (Louis et al., 2022; GINA, 2024). Despite high levels of medication adherence reported in this study, poor asthma control persisted, suggesting the need for further personalized education and treatment adjustments. Frequent exacerbations and hospitalizations highlight the ongoing burden of uncontrolled asthma (GINA, 2024; Beasley et al., 2020).

Several factors, including age, education level, marital status, retirement, and family history of asthma, did not significantly influence asthma control. However, older and retired participants may face additional challenges in managing asthma due to comorbidities and limited healthcare access (Louis et al., 2022). Although education levels did not directly impact control, they may affect patients’ understanding and management of their illness. Lower physical activity among retirees could also negatively affect asthma control.

The large sample size and the lack of similar studies add to the strength of this research. Religious coping explained 15.4% of asthma control, and combined predictors accounted for 56.9%, a remarkably high percentage. Consistent with these findings, the influence of religious coping on disease management and psychological well-being has been observed across various chronic conditions. For instance, Taskin Yilmaz et al. (2022) reported a significant association between positive religious coping and greater satisfaction with life in COPD patients, explaining 38% of the variance. Similarly, Karatana and Yıldız (2024) identified a positive correlation between religious coping, spirituality, and disease self-management in Parkinson’s patients, emphasizing its holistic contributions. Studies on diabetes provide additional evidence; Celik et al. (2022) found that positive religious coping accounted for 7% of the variance in glycemic control, demonstrating its potential to support biological health outcomes.

Adams et al. (2011) highlighted the significance of incorporating psychosocial factors, such as religious coping, into patient care to enhance outcomes in managing chronic diseases. These findings are consistent with the broader body of evidence demonstrating that religious and spiritual coping mechanisms play a vital role in promoting adaptation and emotional resilience among individuals with chronic illnesses. While the contribution of religious coping to asthma control is noteworthy, its impact appears to differ across various diseases, likely due to variations in disease characteristics, cultural contexts, and coping demands. To gain a deeper understanding, further research is warranted to investigate these differences and uncover the underlying mechanisms by which religious coping influences chronic disease management and health outcomes.

The role of nurses is particularly significant in promoting positive religious coping strategies, which have been shown to improve asthma symptoms (Ruppe et al., 2024). Nurses can organize educational programs and provide ongoing follow-up to ensure effective asthma management. By fostering a trusting relationship, they can also encourage the use of positive religious coping as a complementary approach, helping patients find meaning and resilience in managing their condition. For instance, nurses can guide patients in utilizing spiritual resources, such as prayer, mindfulness, or religious community support, as part of their overall care plan.

Healthcare providers, particularly nurses, play a pivotal role in guiding, educating, and supporting asthma patients in managing their condition through proper medication use, lifestyle modifications, and effective coping strategies. Consistent psychosocial support from nurses and other healthcare professionals is crucial in addressing the multifaceted challenges of chronic diseases such as asthma. Adopting a multidisciplinary approach that integrates medical treatment, education, and psychosocial care is essential to achieving improved health outcomes in asthma management. This comprehensive approach should prioritize the development and implementation of psychosocial interventions that address patients’ emotional and spiritual needs in conjunction with their physical health. For instance, healthcare providers can collaborate with chaplains or spiritual counselors to design personalized programs that integrate medical care with religious coping strategies, fostering holistic well-being and more effective disease management.

Furthermore, nurses are in a unique position to assess and address the individual psychosocial factors influencing asthma control. They can incorporate religious coping strategies into routine patient education by emphasizing their potential benefits for stress reduction and symptom management. Such interventions may include group-based discussions on spirituality, one-on-one counseling sessions focusing on religious coping, or referrals to community-based spiritual support services. Expanding these efforts can not only improve asthma management but also enhance patients’ overall quality of life.

To fully realize the potential of integrating religious coping strategies into asthma care, future research should explore how these interventions can be standardized and adapted for diverse populations. By providing detailed, evidence-based guidelines, healthcare providers, particularly nurses, can effectively incorporate these strategies into comprehensive asthma management programs. This holistic approach would ensure that both the biological and psychosocial dimensions of asthma are addressed, leading to more sustainable health outcomes.

## Limitations

The fact that this study is cross sectional and single-center in design limits the ability to establish cause-and-effect relationships, as it reflects only patients attending outpatient clinics at a single hospital. This design may restrict the generalizability of the findings and introduce potential selection bias. Furthermore, the reliance on self-reported data increases the likelihood of response bias among participants. Measuring a subjective concept like religious coping may also yield varying results due to individual differences in religious beliefs and practices, potentially affecting the consistency of the findings.

Additionally, the single-center design may introduce cultural biases specific to the population studied, which should be considered as a potential limitation. The absence of an investigation into comorbidities that could influence asthma control further constrains the scope of the findings.

Future research should address these limitations by employing longitudinal designs to explore causal relationships and expanding the scope to include diverse cultural and religious contexts. Such studies would provide a more comprehensive understanding of how religious coping influences asthma management across different populations and healthcare settings.

## Conclusions

This study revealed that the majority of participants had uncontrolled asthma, with none achieving full control, underscoring the persistent global challenge of effective asthma management. Notably, the findings demonstrated that religious coping strategies, particularly positive religious coping, had a significant impact on asthma control, emphasizing the importance of psychosocial factors in the management of chronic diseases.

These results highlight the critical need for a multidisciplinary approach to asthma care that integrates medical treatment, patient education, and psychosocial support. Nurses are central to this model, providing guidance on medication adherence, trigger management, and the adoption of healthy lifestyle practices. Beyond addressing physical needs, fostering positive religious coping strategies and offering psychosocial support are essential for improving asthma outcomes, particularly in adults with chronic conditions. Such approaches are vital for developing personalized interventions that enhance both the quality of life and overall health outcomes for adults living with asthma.

## Data Availability

The data that support the findings of this study are available on request from the corresponding author. The data are not publicly available due to privacy or ethical restrictions.
